# Ultra-low-field magneto-elastocaloric cooling in a multiferroic composite device

**DOI:** 10.1038/s41467-018-06626-y

**Published:** 2018-10-04

**Authors:** Huilong Hou, Peter Finkel, Margo Staruch, Jun Cui, Ichiro Takeuchi

**Affiliations:** 1 Department of Materials Science and Engineering, University of Maryland, College Park, MD 20742 USA; 2 Materials Science and Technology Division, U.S. Naval Research Laboratory, Washington, DC 20375 USA; 3 Division of Materials Science and Engineering, Ames Laboratory, Ames, IA 50011 USA; 4 Department of Materials Science and Engineering, Iowa State University, Ames, IA 50011 USA

## Abstract

The advent of caloric materials for magnetocaloric, electrocaloric, and elastocaloric cooling is changing the landscape of solid state cooling technologies with potentials for high-efficiency and environmentally friendly residential and commercial cooling and heat-pumping applications. Given that caloric materials are ferroic materials that undergo first (or second) order phase transitions near room temperature, they open up intriguing possibilities for multiferroic devices with hitherto unexplored functionalities coupling their thermal properties with different fields (magnetic, electric, and stress) through composite configurations. Here we demonstrate a magneto-elastocaloric effect with ultra-low magnetic field (0.16 T) in a compact geometry to generate a cooling temperature change as large as 4 K using a magnetostriction/superelastic alloy composite. Such composite systems can be used to circumvent shortcomings of existing technologies such as the need for high-stress actuation mechanism for elastocaloric materials and the high magnetic field requirement of magnetocaloric materials, while enabling new applications such as compact remote cooling devices.

## Abstract (web summary)

The broad use of elastocaloric materials in cooling applications is hindered by the need to exert large forces onto the material. Compressing a magnetostrictive-elastocaloric composite using a low magnetic field of 0.16 T, temperature changes up to 4 K are achieved without applying external forces.

## Introduction

Elastocaloric cooling exploiting the stress-induced martensitic phase transformation of shape memory alloys (SMAs) has recently emerged as a strong alternative cooling technology candidate due to the intrinsically high coefficient of performance of elastocaloric materials^1–5^. Compared to other solid-state cooling techniques, its potentials for high-efficiency cooling systems are only rivaled by magnetocaloric cooling^6^. Previously, compression-based 400 W systems and tension-based device and active regenerators have been demonstrated using the elastocaloric effect^7–10^. Despite its high efficiency, one disadvantage of elastocaloric cooling is the large stress required to induce the martensitic transformation. For a commonly available Ni-Ti SMA, for instance, >600 MPa is required in compression for the transformation^11,12^. There are only a handful of engineering options for exerting such large stress, making it challenging to design compact cooling devices.

There have been many demonstrations of composite multiferroic effects that take place via elastic coupling between magnetostrictive and piezoelectric materials at their interfaces, and they have been explored for a variety of bulk and thin-film device applications including ultra-high-sensitivity magnetic field sensors^13–16^, cantilever-based mechanical logic devices^17,18^, and voltage-controlled nanoscale magnetic domain memories^19^. They take advantage of mechanical transduction through strain transfer between materials of similar Young’s moduli^20^.

In this work, we demonstrate the utility of multiferroic cooling devices enabled by elastic coupling of a magnetostrictive material with a superelastic SMA for the first time. In particular, we employ magnetostrictive strain to induce elastocaloric cooling in a composite configuration. Our multiferroic devices consist of Tb_*x*_Dy_1−*x*_Fe_2_ (*x* ~ 0.3, Terfenol-D), which can provide strain with load stress as large as 880 MPa^21^ and a Copper–Aluminum–Manganese (Cu–Al–Mn) SMA whose adiabatic temperature change, Δ*T*_ad_, can be as large as 12.8 K^22^. The magnetic-field-induced elastocaloric (which we call magneto-elastocaloric (M-eC) for short) cooling devices achieve cooling Δ*T*_ad_ of 4 K with 0.16 T, which can open up possibilities for an entirely new class of compact and remote cooling applications.

## Results

### Magneto-elastocaloric pathway using multiferroic composites

**Fig. 1:**
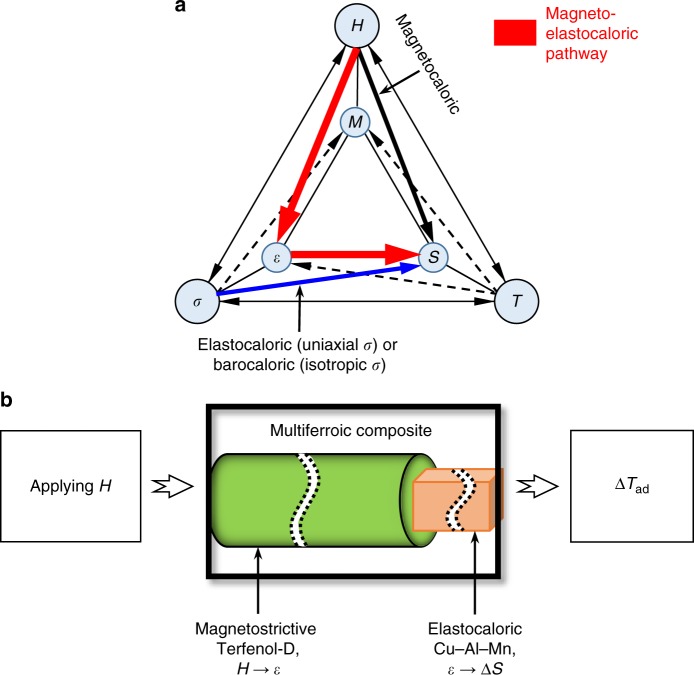
Magneto-elastocaloric multiferroic composite. **a** A modified Heckmann diagram illustrating the pathway leveraging magnetic field for cooling in a multiferroic composite. Symbols: magnetic field (*H*), magnetization (*M*), stress (*σ*), strain (*ε*), temperature (*T*), and entropy (*S*). Red arrow is the composite magneto-elastocaloric pathway demonstrated in this work, while black and blue arrows are intrinsic magnetocaloric and mechanocaloric (elastocaloric under uniaxial *σ* or barocaloric under isotropic *σ*) pathways, respectively. **b** Schematic of the magneto-elastocaloric (M-eC) device, in which a magnetostrictive material (Terfenol-D) and a single crystal Cu–Al–Mn shape memory alloy (SMA) are elastically coupled to generate cooling under magnetic field. Terfenol-D displays extension when magnetic field is applied along the length of Terfenol-D and retraction upon removal of the magnetic field. The SMA generates an isothermal entropy change at a small strain rate and an adiabatic temperature change at a large strain rate through elastocaloric effect

The functionality of our composite multiferroic devices corresponds to the red arrow path in the modified Heckmann diagram (Fig. 1a), which includes temperature and entropy as a field and a conjugate response parameter, and it is effectively an alternative route to achieve the magnetocaloric effect (black arrow). A well-known issue of intrinsic magnetocaloric materials is the relatively large magnetic field they require to achieve adiabatic cooling. For instance, magnetic field as large as 2 T is needed to induce Δ*T*_ad_ of 5.4 K in gadolinium^23^. In contrast, we use Terfenol-D whose magnetostrictive strain can be as large as 2000 ppm at <1 T to mechanically load single crystal Cu–Al–Mn SMA, which undergoes transformation with a relatively small stress of ≈100 MPa.

The schematic of the M-eC device is shown in Fig. 1b. The frame of the device acts as fixed constraints against the overall extension of the multiferroic composite so that mechanical load is transferred from Terfenol-D to Cu–Al–Mn SMA. We use a tabletop electromagnet to generate magnetic field, and a Lakeshore Hall probe is used to measure its magnitude at the surface of Terfenol-D. The temperature change of the Cu–Al–Mn SMA piece in the M-eC device is measured by an infrared camera.

### Cooling by exploiting low magnetic fields

**Fig. 2:**
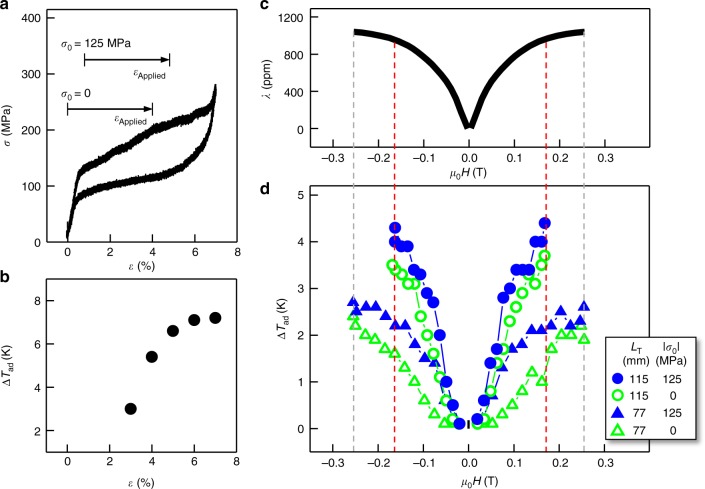
Tailoring low-field adiabatic cooling. **a**, **b** Compressive stress, *σ*, at slow loading-unloading (**a**) and measured cooling Δ*T*_ad_ upon rapid unloading (**b**) in the Cu–Al–Mn shape memory alloy (SMA) piece as a function of strain, *ε*, tested in a servohydraulic load frame. **c**, **d** Magnetostrictive strain, *λ*, of the Terfenol-D (**c**) and cooling Δ*T*_ad_ of the Cu–Al–Mn SMA (**d**) in the magneto-elastocaloric (M-eC) device as a function of magnetic field, *μ*_0_*H* (which is removed rapidly for cooling) for two different pre-stresses, *σ*_0_, applied to the SMA for two different lengths, *L*_T_, of Terfenol-D used in the device. The pre-stress, *σ*_0_, signifies the stress level at the onset of applying the strain from magnetostriction of Terfenol-D, *ε*_Applied_, to SMA as indicated by arrows in **a**. Magnetostriction of Terfenol-D at various pre-loaded stresses is shown in Supplementary Figure 1. The dashed lines denote the correspondence of Δ*T*_ad_ and *λ* for the same applied field in the M-eC device

We first investigate the basic properties of components of the M-eC device and then characterize the cooling of the composite device. A typical stress–strain curve of the Cu–Al–Mn SMA piece measured in a conventional servohydraulic load frame is shown in Fig. 2a. The strain is fully recoverable, and it is the transformation volume fraction that determines the released (and absorbed) latent heat, which in turn controls the ultimate cooling Δ*T*_ad_ in the SMA. Different Δ*T*_ad_ (Fig. 2b) are attained by rapidly unloading from different strain levels. Terfenol-D is known to display a saturated magnetostrictive strain up to 2000 ppm^13^. Here we focus on the low-magnetic-field effect: it shows a magnetostriction of 930 ppm at 0.16 T (Fig. 2c), which can be used to attain Δ*T*_ad_ in the M-eC device as large as 4.4 K (Fig. 2d). A Δ*T*_ad_ of 4.4 K corresponds to a strain of 3.7% in the SMA according to Fig. 2a, b, which is smaller than the directly measured strain (~5%) in the device due to magnetostriction at 0.16 T (Fig. 2c). We have estimated contribution to the strain due to the compliance of the device frame to be about 1%, which can account for the difference (See Methods section for detailed calculations). We expect to be able to obtain larger Δ*T*_ad_ by incorporating a more rigid device frame in the future. Out of different Terfenol-D lengths and pre-stress configurations we have looked at (Fig. 2d), a longer Terfenol-D piece naturally gives rise to a larger strain, and a pre-loaded stress to the Cu–Al–Mn piece also leads to a larger strain, resulting in larger observed Δ*T*_ad_.

**Fig. 3:**
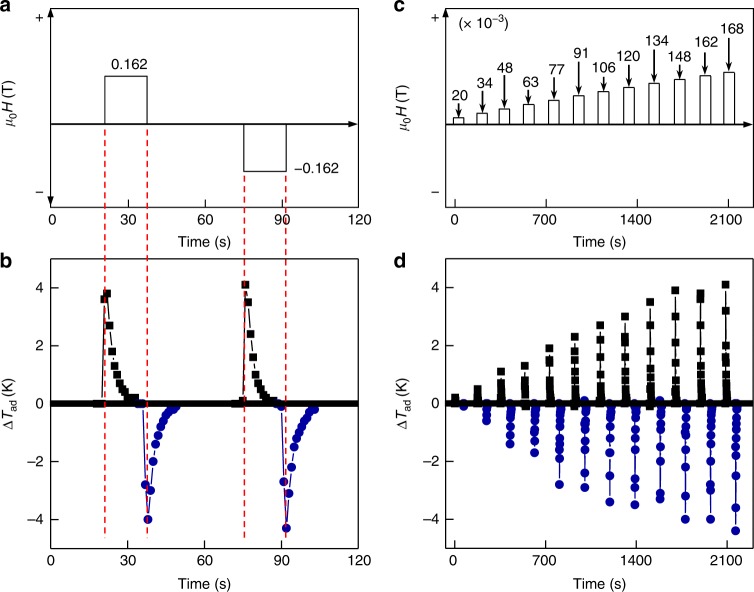
Adiabatic temperature change under low magnetic fields. **a**, **b** Waveform of applied magnetic field, *μ*_0_*H* (**a**) and corresponding adiabatic temperature change, Δ*T*_ad_, of the Cu–Al–Mn shape memory alloy (SMA) in the magneto-elastocaloric (M-eC) device (**b**) under rapid application and removal of positive and negative magnetic fields. Black squares indicate the heating part of the data and blue circles mark the cooling part of the data. The red dashed lines in **a** and **b** indicate that measured heating Δ*T*_ad_ and cooling Δ*T*_ad_ are in direct response to the magnetic field change. **c**, **d** Increasing magnitude of magnetic field (**c**) and resulting increase in heating and cooling in Cu–Al–Mn SMA (**d**) in the M-eC device

We performed a series of measurements of the low-magnetic-field M-eC effect using a 115-mm-long Terfenol-D rod with a pre-loaded stress of 125 MPa. Rapid application and rapid removal of field with positive and negative values (Fig. 3a) leads to a heating and a cooling, respectively, both followed by a natural settling back to room temperature (Fig. 3b). The magnetic field of 0.02 and 0.168 T (Fig. 3c) results in a cooling Δ*T*_ad_ (nearly the same as heating Δ*T*_ad_) of 0.2 and of 4.4 K, respectively (Fig. 3d). Δ*T*_ad_ increases linearly with increasing magnitude of magnetic field.

### Magnetic-field-dependent cooling strength

**Fig. 4:**
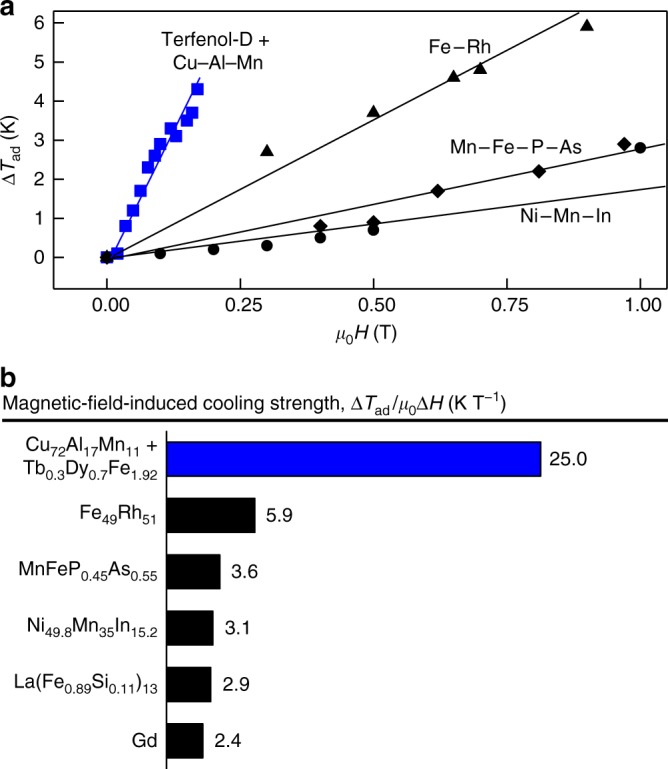
Comparison of low magnetic field cooling. **a** Cooling Δ*T*_ad_ as a function of applied magnetic field, *μ*_0_*H* for selected materials systems. **b** Comparison of magnetic-field-induced cooling strength defined as the ratio of observed Δ*T*_ad_ to the applied magnetic field, *μ*_0_Δ*H*. Selected materials systems with the change of applied magnetic field: Gd (at 1.9–7.5 T)^23^, Fe_49_Rh_51_ (at 0.3–2.5 T)^24^, MnFeP_0.45_As_0.55_ (at 0.4–1.4 T)^25^, Ni_49.8_Mn_35_In_15.2_ (at 0.1–2 T)^26^, and La(Fe_0.89_Si_0.11_)_13_ (at 2 T)^27^. Only directly measured experimental data are listed

Thus these M-eC devices are functionally able to achieve low magnetic-field-induced cooling. For comparison, we plot Δ*T*_ad_ versus applied magnetic field for our devices and selected conventional magnetocaloric materials (Fig. 4a). Our M-eC devices can attain a Δ*T*_ad_ of 4 K with a field of 0.16 T; in contrast, FeRh would require 0.6 T to achieve 4 K, and fields much >1 T are needed for Mn–Fe–P–As and Ni–Mn–In. We define magnetic-field-induced cooling strength to be Δ*T*_ad_/*μ*_0_Δ*H* and use it as a metric of magnetic-field-induced cooling (Fig. 4b). Among various materials systems, Δ*T*_ad_/*μ*_0_Δ*H* of the composite devices here is three times larger than that of FeRh, an intrinsic magnetocaloric material with the highest magnetocaloric magnetic-field-induced cooling strength^28,29^.

### Design of compact cooling devices

**Fig. 5:**
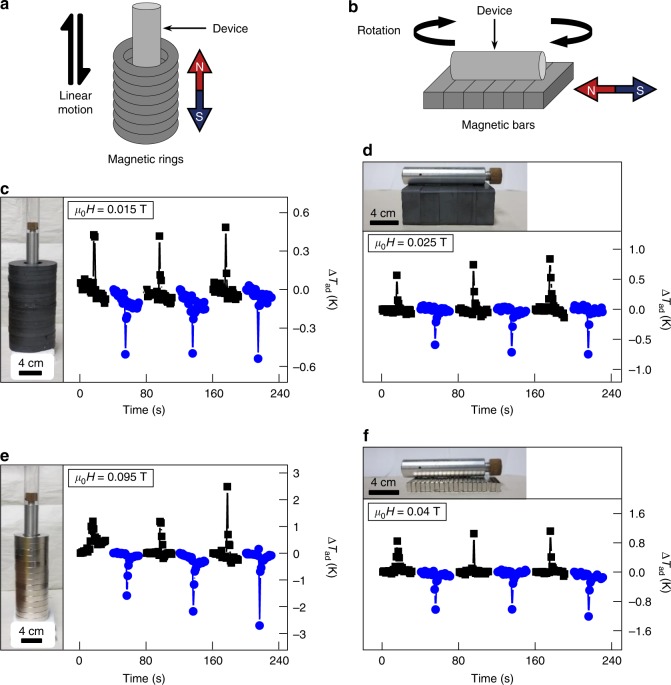
Demonstrations of compact low-magnetic-field-induced cooling. **a**, **b** Motion of magneto-elastocaloric (M-eC) device with respect to the magnetic field from stacked ring (**a**) and bar (**b**) permanent magnets in compact geometries leads to heating and cooling. In schematics **a** and **b**, arrows indicate the movement of the device against the static magnet stacks, and the N/S symbols mark the orientation of the magnetic field. **c**–**f** Directly measured Δ*T*_ad_ (heating in black and cooling in blue) is in response to the magnetic field generated by ferrite magnets (**c**, **d**) and by Nd–Fe–B magnets (**e**, **f**). The typical device speed is ±40 mm s^−1^ for the stacked ring configurations, and 3.5 rad s^−1^ for rotating the device in and out of the field axis in the bar configuration. In **c**–**f**, the insets are photographs of the M-eC device with permanent magnets for size comparison, and the fields are the measured values at the surface of Terfenol-D in the M-eC device

In addition to the high magnetic-field-induced strength, another major advantage of the M-eC devices is their compactness. By enlisting magnetostrictive strain, we have removed cumbersome, large-stress actuation mechanisms to achieve elastocaloric cooling. To illustrate design flexibilities and the straightforward implementation of the M-eC effect, we have demonstrated several geometries where simple relative motion of the device with respect to permanent magnets is used to achieve cooling. In the schematics shown in Fig. 5, the device is inserted in and out of stacked ring magnets (Fig. 5a) or rotated above the plane of magnets in and out of the field axis (Fig. 5b). Figure 5c, d show the resulting heating and cooling achieved using ferrite magnets, and Fig. 5e, f show the magneto-elastocaloric effect achieved with Nd–Fe–B magnets. In all instances, the achieved Δ*T*_ad_ is consistent with the magnetic field (insets of Fig. 5c–f) measured at the surface of Terfenol-D in the M-eC device.

## Discussion

The demonstrations with the permanent magnets indicate that, with proper designs, the M-eC devices can be even more compact. By implementing a design where the permanent magnet is placed even closer to the surface of the Terfenol-D piece, we would be able to attain larger magnetostriction and consequently larger Δ*T*_ad_ in the Cu–Al–Mn piece. As an alternative to magnetostriction (magnetic-field-induced strain), it is also possible to use piezoelectric materials to construct another type of multiferroic composites for inducing elastocaloric cooling. In particular, recent development in advanced piezoelectric single crystals^30,31^ indicates that compact piezoelectric/superelastic SMA composite is a promising option for achieving the electric-field-induced elastocaloric effect or the electro-elastocaloric effect. A similar electric-field-induced mechanocaloric effect concept has been previously mentioned^32^.

We note that functionally our magnetic-field-induced elastocaloric effect (which we call magneto-elastocaloric effect) is fundamentally different from the previously reported multicaloric effects in intrinsic materials such as FeRh and Ni–Mn–Sn(Cu) where magnetic field and stress are both applied to the material to induce Δ*S*^33,34^.

The efficiency of magnetostrictive Terfenol-D is typically measured at a resonant frequency (~500 Hz), and it can potentially be 50–70%^13^. In this work, Terfenol-D is used at a much lower frequency, and we believe its efficiency is ≈20% based on references on similar materials^13,35^. We have previously reported on the high efficiency (materials coefficient of performance) of elastocaloric SMAs^2^. The high efficiency, however, is contingent on being able to implement a system incorporating work recovery. We have previously demonstrated work recovery in elastocaloric cooling systems by stacking two sets of SMAs linearly and operating the sets in a reciprocating manner^7,36^. In such previous embodiments, actuators are used to facilitate loading/unloading involved in work recovery. While yet to be physically demonstrated herein, it is straightforward to extend the same actuation design: we would replace an actuator with a pair of aligned Terfenol-D pieces (one on each side of a mechanical load), and by applying the magnetic field on and off in an alternating manner to the pair, work recovery can be implemented.

We envision M-eC devices being deployed for a variety of remote and compact applications including cooling of electronic components, photon detectors, sonar sensors, and micro-refrigerators. An intriguing possibility is to use it for local brain cooling to treat epileptic seizures^37,38^. We believe the non-contact, wireless nature of the compact magnetostrictive–SMA composite can be competitive in many application arenas where miniature Peltier cooling devices currently dominate the market. It is important to note that our devices can be operated in the cooling-only mode under isothermal loading and adiabatic unloading (by slowly increasing the magnetic field followed by rapid removal as shown in Supplementary Figure 2) to curtail heating. For these local cooling applications, the key is not to raise the temperature of the surrounding (the body of the device/material) during loading. The loading speed that can keep the device and the environment/reservoir isothermal depends on the heat capacity of the surrounding. Alternatively, the device can be used in the single shot at a time mode, where loading part of the cycle is performed elsewhere beforehand, and the device is then placed at a location to be cooled, ready to deliver adiabatic cooling as described in a U.S. patent^39^. Naturally, such an operation mode is desirable for a multitude of localized cooling applications.

We also envision our devices to be configured in an array geometry to operate in more conventional cooling applications together with heat exchangers and other standard accessories including a reciprocating work recovery mechanism. In such systems, exothermic heat would be handled in a standard heat-rejection part of the cycle. While the detailed analysis of such proposed systems is outside the scope of the current work, we believe the unique design of our device would again enable compact applications with no need for large actuators^7^.

For long-term operation of devices and systems, fatigue life is a concern. Generally speaking, Cu-based SMAs are not as good as NiTi with regard to their fatigue behavior. There are a number of strategies that can be implemented for extending their fatigue life^7^: although they require a more elaborate synthesis process, single crystal Cu–Al–Mn alloys have good fatigue resistance compared to polycrystalline counterparts^40^; a compressive mode is known to lead to substantially extended fatigue life compared to when tension is used; and applying smaller strains is able to extend fatigue life. We are currently performing a long-term fatigue test on our Cu–Al–Mn single crystal piece. To date, after ~10,000 cycles under compression (with a strain of 4%), we have observed minimal sign of fatigue in the cooling behavior^7^. For the remote cooling device applications mentioned above, 10,000 cycles are more than sufficient. For conventional cooling systems applications, the cycle number required is in millions, numbers recently observed in NiTi thin films^41^ as well as in bulk NiTi^42^. With proper microstructure and composition control as well as operational practices, we believe it is also possible to develop Cu-based SMAs for extended operation in the near future.

## Methods

### Fabrication and characterization of materials

Cu–Al–Mn alloys with a nominal composition of Cu_72_Al_17_Mn_11_ (at.%) were prepared by induction heating of elemental powders with a purity of 99.9 at.% followed by abnormal grain growth via thermal processing to attain single crystal specimens. Details of the preparations are available in previous publications^43,44^. The single crystalline structure was confirmed by X-ray diffraction, and the composition was determined using wavelength dispersive spectroscopy with calibrated standards. The transformation temperatures were analyzed by differential scanning calorimetry (DSC Q100, TA Instrument Inc.), and they were found to be *M*_s_= 271 ± 1 K, *M*_f_= 251 ± 1 K, *A*_s_= 270 ± 1 K, and *A*_f_= 283 ± 1 K. The latent heat was found to be Δ*H*_A→M_= 4.1 J g^−1^ and Δ*H*_M→A_= 5.3 J g^−1^. Standard stress–strain tests were carried out on a MTS 810 servohydraulic load frame at a strain rate of 0.0002 s^−1^ for isothermal loading–unloading and at a loading strain rate of 0.0002 s^−1^ and an unloading strain rate of 5 s^−1^ for adiabatic cooling.

Terfenol-D alloy (purchased from ETREMA Products, currently TdVib LLC) had a composition of Tb_0.3_Dy_0.7_Fe_1.92_. The linear magnetostrictive strain of the Terfenol-D was estimated to be 800–1200 ppm. In this work, two Terfenol-D rods with a diameter of 6 mm were machined by electrical discharge machining, one with the length of 77 mm and the other with the length of 38 mm. Both ends of each Terfenol-D rod were fine cut for obtaining smooth surfaces to mechanically interface with Cu–Al–Mn SMA for assembling a multiferroic composite.

To house the multiferroic composite, a high-strength aluminum frame was customized with an outer diameter of 28.0 mm and an inner diameter of 19.2 mm with two ends capped with Brass knobs, which can be tightened/untightened by an Allen wrench for adding/reducing the pre-stress load to the multiferroic composite. A polyimide ring inside the frame was used to guide the multiferroic composite for avoiding lateral deformation, and ceramic disks were inserted to insulate Cu–Al–Mn SMA from surrounding thermal mass.

The load generated by the Terfenol-D was sufficient to actuate the Cu–Al–Mn SMA. We estimated the 115-mm-long Terfenol-D rod (with a diameter of 6 mm and a cross-sectional area of 28.2 mm^2^) produced a load of 300 N under the constraint by the frame of the M-eC device (Fig. 1b). This load was able to initiate the phase transformation in the Cu–Al–Mn SMA piece in the M-eC device with a transformation stress of ≈100 MPa, which required the cross-sectional area of the SMA specimen to be ≈3 mm^2^ at most. We thus used rectangular Cu–Al–Mn specimens with 2 mm × 1 mm × 2 mm in dimensions: the cross-sectional area that came in contact with a Terfenol-D rod was 2 mm^2^, and the 2-mm length was along the crystallographic orientation [110]. When the Terfenol-D was placed with Cu–Al–Mn under a preload of 300 N, we characterized the magnetostriction of Terfenol-D with an attached strain gauge read by a strain indicator (Vishay Measurements Model P-3500) to be 930 ppm at 0.16 T (see Supplementary Figure 1). In our M-eC device, we were able to achieve a displacement of 0.106 mm from the 115-mm-long Terfenol-D rod, which would have generated 5.3% compressive strain in the Cu–Al–Mn piece. We noted, however, under the working load cycle of 0−300 N, that there was mechanical compliance in various components of the device frame, which consisted of parts made of aluminum and brass, given their finite rigidity. For instance, an aluminum fixture in the device under this load was expected to experience deformation that was equivalent to a fraction of a percent in the strain in the Cu–Al–Mn piece. Other compliance contributions of similar order were also expected from bolts and threads, as well as non-linear response of contact surfaces within the device. Together, we expected the overall compliance of the device frame to be ⪆ 1% in equivalent strain to the SMA piece, accounting for the observed difference in the strain between 5.3% and 3.7%.

### Electromagnet setting and in situ thermal imaging

Magnetic fields were generated and precisely controlled by an H-frame electromagnet (Micro-Now Instrument Inc.) equipped with a power supply (Model BOP 50-20 MG, Korea Electric Power Corporation) at a maximum output of 50 V and 20 A. The center of the pole caps of the electromagnets was aligned to the longitudinal axis of the composite device, and the space between the pole caps was set to exactly fit the device without gaps to attain highest possible fields to be experienced by the Terfenol-D rods in the device. The magnetic fields were measured using a Lakeshore Hall probe placed in the middle of the device.

During application and removal of magnetic field, the temperature of Cu–Al–Mn piece in the device was directly monitored using an infrared camera (T450sc, FLIR Systems, Inc.) by collecting thermal videos at a frame rate of 10 Hz, a spatial resolution of 0.00136 rad, and a thermal sensitivity of 0.03 K at 303 K after calibrations with real-time temperature. We compared measurements of temperature changes at 10 Hz frame rate and higher frame rate, and we found that there was no observable difference as shown in Supplementary Figure 3. A spot meter detected the temperature for an area of 2 × 2 mm^2^ on the Cu–Al–Mn piece in the device during recording of videos, from which thermal images of 320 × 240 pixels were extracted for time-wise analysis of the temperature change. The temperatures were averaged over 3-by-3 pixels at a distance of ~0.4 m to focus on the 2-by-2 mm^2^ area of the single crystal Cu–Al–Mn piece (see Supplementary Figure 4). A thin coating of graphite with an emissivity coefficient of 0.95 was sprayed on the surface of Cu–Al–Mn SMA to increase its thermal emissivity. The procedure of recording, extracting, and analyzing was repeated for each combination of the experimental parameters, including magnetic field, preloaded stress, and length of Terfenol-D.

### Permanent magnet setting and motion-related cooling

Commercially available permanent magnets (ring and bar magnets of ferrite and Nd–Fe–B) were used as the source of low magnetic field. The gray ferrite and the silver Nd–Fe–B ring magnets had an outer diameter of 115.3 and 76.2 mm, an inner diameter of 44.5 and 38.1 mm, and a thickness of 10.2 and 12.7 mm, respectively. The gray ferrite and the silver Nd–Fe–B bar magnets had a length of 76.2 and 76.2 mm, a width of 50.8 and 12.7 mm, and a thickness of 25.4 and 6.4 mm, respectively, and they were stacked vertically or horizontally. A calibrated thermocouple was used to record the temperature of the SMA piece.

## Electronic supplementary material

Supplementary Information

## Data Availability

The data that support the findings of this study are available from the corresponding author upon reasonable request.
