# Supplementary material for: Ultra-low-field magneto-elastocaloric cooling in a multiferroic composite device
Source: Nat Commun. 2018 Oct 4;9:4075. doi: 10.1038/s41467-018-06626-y (PMC6172219; doi:10.1038/s41467-018-06626-y)
Supplement: Supplementary file 1 — Supplementary Information [file 41467_2018_6626_MOESM1_ESM.pdf]

Supplementary Information

Ultra-low-field magneto-elastocaloric cooling in a multiferroic composite device

Hou et al.

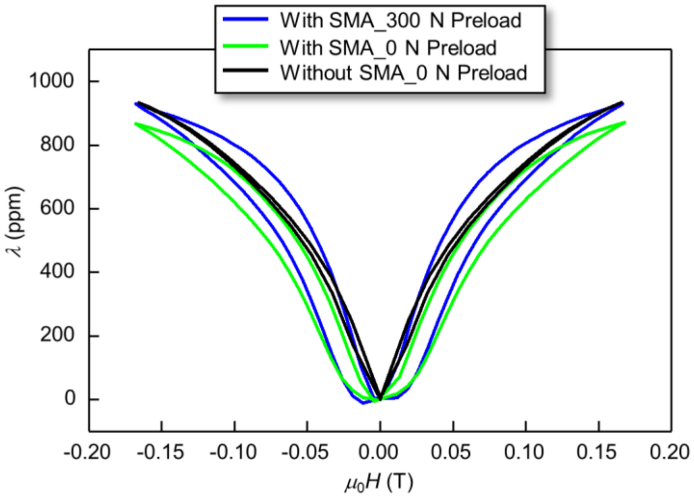

Supplementary Figure 1. Magnetostriction of Terfenol-D. The magnetostriction of the Terfenol-D as a function of magnetic field is measured when the Terfenol-D is placed with and without Cu–Al–Mn shape memory alloy (SMA), and with and without a pre-load (of 300 N) for the case with Cu–Al–Mn SMA.

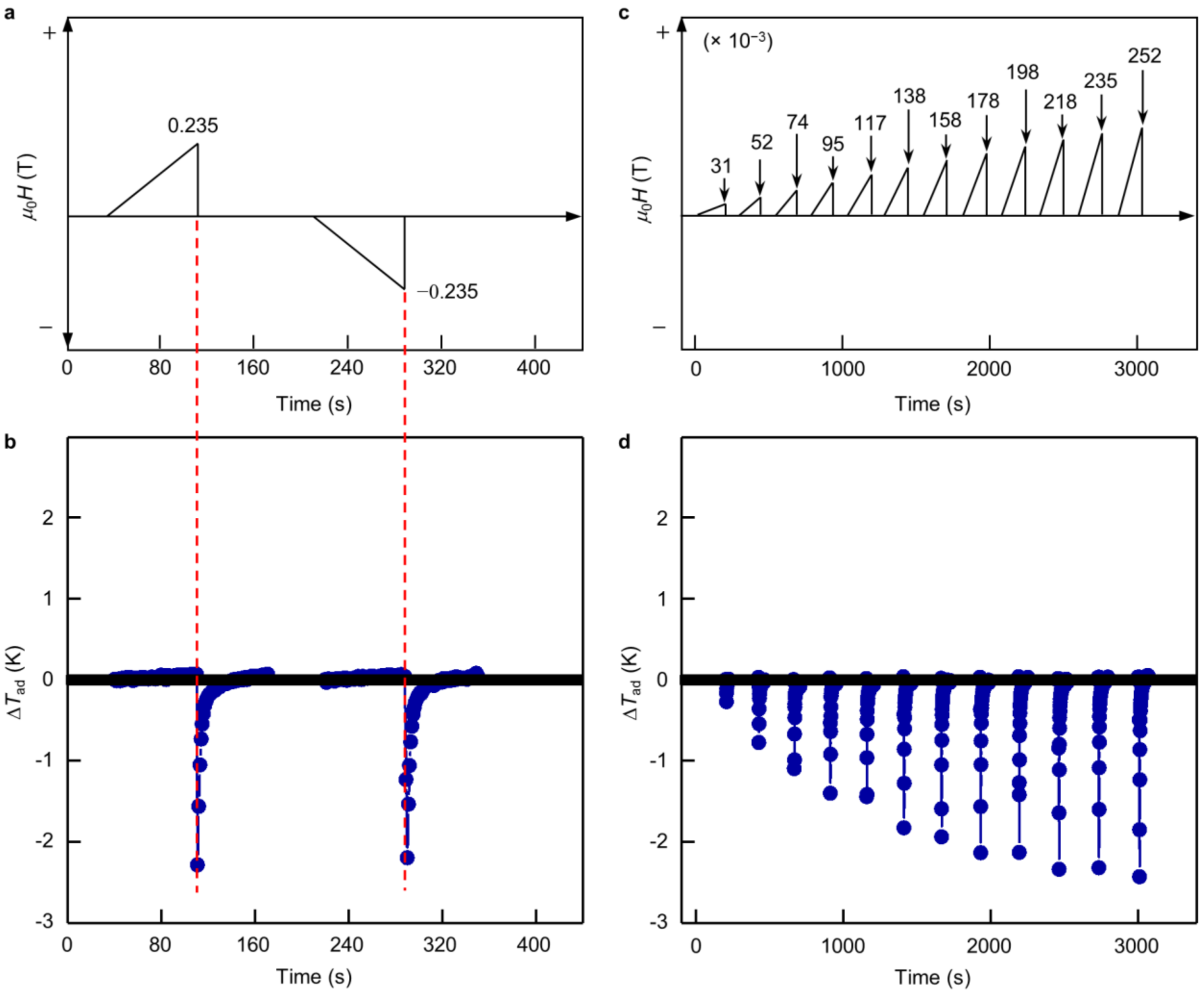

Supplementary Figure 2. Cooling without self-heating of elastocaloric materials. a,**b**, Waveform of applied magnetic field, $\mu_0 H$ (**a**) and corresponding adiabatic temperature change, ΔT_{ad}, in the Cu–Al–Mn shape memory alloy (SMA) in the magneto-elastocaloric (M-eC) device (**b**) under slow ramp up and rapid removal of positive and negative magnetic fields. The red dashed lines in **a** indicate that measured cooling part of ΔT_{ad} are in direct response to the rapid change of magnetic field. **c**,**d**, Increasing magnitude of maximum magnetic field (**c**) and resulting increase in cooling ΔT_{ad} in SMA (**d**).

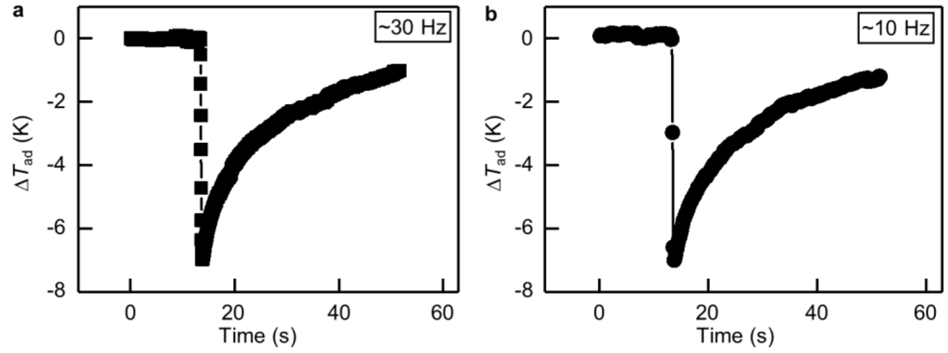

Supplementary Figure 3. Comparison of data collected at different frequencies. a,**b**, The same Cu–Al–Mn piece is tested using the same strain and strain rate at sampling frequencies of 30 Hz (**a**) and at 10 Hz (**b**) on the FLIR T450sc camera. There is no observable difference in cooling ΔT_{ad}.

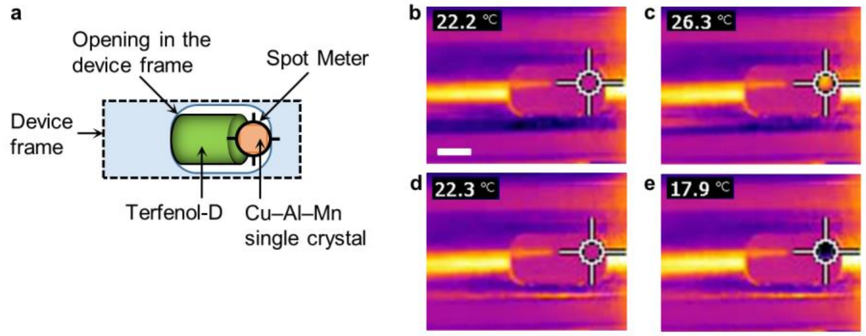

Supplementary Figure 4. Measurement setup and representative thermal images. a, Schematic showing a 2 mm × 2 mm Cu–Al–Mn shape memory alloy (SMA) piece placed inside the spot meter of the camera view at an object-to-camera distance of 0.4 m. **b–e**, A typical measurement cycle: at initial equilibrium point (**b**), after rapid increase of magnetic field (**c**), after the field is held, and equilibrium is reached (**d**), and upon adiabatic removal of magnetic field (**e**). In **b–e**, the temperature reading of the single crystal Cu–Al–Mn SMA by the spot meter is at the top-left corner. Scale bar in **b** is 5 mm.
